# Global epidemiology and clinical outcomes of carbapenem-resistant *Pseudomonas aeruginosa* and associated carbapenemases (POP): a prospective cohort study

**DOI:** 10.1016/S2666-5247(22)00329-9

**Published:** 2023-02-09

**Authors:** Jinnethe Reyes, Lauren Komarow, Liang Chen, Lizhao Ge, Blake M Hanson, Eric Cober, Erica Herc, Thamer Alenazi, Keith S Kaye, Julia Garcia-Diaz, Lanjuan Li, Souha S Kanj, Zhengyin Liu, Jose M Oñate, Robert A Salata, Kalisvar Marimuthu, Hainv Gao, Zhiyong Zong, Sandra L Valderrama-Beltrán, Yunsong Yu, Paul Tambyah, Gregory Weston, Soraya Salcedo, Lillian M Abbo, Qing Xie, Karen Ordoñez, Minggui Wang, Martin E Stryjewski, Jose M Munita, David L Paterson, Scott Evans, Carol Hill, Keri Baum, Robert A Bonomo, Barry N Kreiswirth, Maria Virginia Villegas, Robin Patel, Cesar A Arias, Henry F Chambers, Vance G Fowler, Yohei Doi, David van Duin, Michael J Satlin

**Affiliations:** Molecular Genetics and Antimicrobial Resistance Unit, Universidad El Bosque, Bogotá, Colombia (J Reyes PhD); The Biostatistics Center, George Washington University, Rockville, MD, USA (L Komarow MS, L Ge MAS, S Evans PhD); Center for Discovery and Innovation and Department of Medical Sciences, Hackensack Meridian School of Medicine, Nutley, NJ, USA (L Chen PhD, B N Kreiswirth PhD); Center for Infectious Diseases and Microbial Genomics, UTHealth, McGovern School of Medicine at Houston, Houston, TX, USA (B M Hanson PhD); Department of Infectious Diseases, Cleveland Clinic, Cleveland, OH, USA (E Cober MD); Division of Infectious Diseases, Henry Ford Hospital, Detroit, MI, USA (E Herc MD); College of Medicine, King Abdulaziz Medical City, Riyadh, Saudi Arabia (T Alenazi MD); Division of Infectious Diseases, University of Michigan, Ann Arbor, MI, USA (K S Kaye MD); Division of Allergy, Immunology, and Infectious Diseases, Rutgers Robert Wood Johnson Medical School, New Brunswick, NJ, USA (K S Kaye); Division of Infectious Diseases, Ochsner Medical Center, New Orleans, LA, USA (J Garcia-Diaz MD); State Key Laboratory for Diagnosis and Treatment of Infectious Diseases, The First Affiliated Hospital of Medical School of Zhejiang University, Hangzhou, China (L Li MD); Division of Infectious Diseases, American University of Beirut Medical Center, Beirut, Lebanon (S S Kanj MD); Infectious Disease Section, Department of Internal Medicine, Peking Union Medical College Hospital, Beijing, China (Z Liu MD); Servicio de Medicina Interna, Centro Medico Imbanaco, Cali, Colombia (J M Oñate MD); Department of Medicine, Case Western Reserve University School of Medicine, Cleveland, OH, USA (R A Salata MD); Department of Infectious Diseases, Tan Tock Seng Hospital, National Centre for Infectious Diseases, Singapore (K Marimuthu MBBS); Department of Infectious Diseases, Shulan Hangzhou Hospital, Hangzhou, China (H Gao MD); Center of Infectious Diseases, West China Hospital of Sichuan University, Chengdu, China (Z Zong MD); Infectious Diseases Research Group, School of Medicine, Hospital Universitario San Ignacio, Pontificia Universidad Javeriana, Bogotá, Colombia (S L Valderrama-Beltrán MD); Department of Infectious Diseases, Sir Run Shaw Hospital, Zhejiang University School of Medicine, Hangzhou, China (Y Yu PhD); National University of Singapore, Infectious Diseases Translational Research Program, Singapore (P Tambyah MD); Division of Infectious Diseases, Department of Medicine, Montefiore Medical Center, Albert Einstein College of Medicine, Bronx, NY, USA (G Weston MD); Servicio de Infectología, Organizacion Clinica General del Norte, Barranquilla, Colombia (S Salcedo MD); Division of Infectious Diseases, University of Miami Hospital, Miami, FL, USA (L M Abbo MD); Department of Infectious Disease, Ruijin Hospital, Shanghai, China (Q Xie MD); Department of Infectious Diseases, ESE Hospital Universitario, San Jorge de Pereira, Pereira, Colombia (K Ordoñez MD); Institute of Antibiotics, Huashan Hospital, Fudan University, Shanghai, China (M Wang MD); Department of Medicine and Division of Infectious Diseases, Centro de Educación Médica e Investigaciones Clínicas, Buenos Aires, Argentina (M E Stryjewski MD); Millennium Initiative for Collaborative Research on Bacterial Resistance, Instituto de Ciencias e Innovación en Medicina, Facultad de Medicine, Clínica Alemana, Universidad del Desarrollo, Santiago, Chile (J M Munita MD); Department of Infectious Diseases, University of Queensland Centre for Clinical Research, Royal Brisbane and Women’s Hospital, Brisbane, QL, Australia (D L Paterson MD); Duke Clinical Research Institute, Duke University, Durham, NC, USA (C Hill PhD, K Baum BS, V G Fowler Jr MD); Case Western Reserve University-Veteran Affairs Center for Antimicrobial Resistance and Epidemiology, Research Service, Louis Stokes Cleveland Department of Veterans Affairs Medical Center, Cleveland, OH, USA (R A Bonomo MD); Department of Medicine, Pharmacology, Molecular Biology and Microbiology, and Biochemistry, Case Western Reserve University School of Medicine, Cleveland, OH, USA (R A Bonomo); Grupo de Resistencia Antimicrobiana y Epidemiología Hospitalaria, Universidad El Bosque, Bogotá, Colombia (M V Villegas MD); Division of Clinical Microbiology, Department of Laboratory Medicine and Pathology and Division of Infectious Diseases, Department of Medicine, Mayo Clinic, Rochester, MN, USA (R Patel MD); Division of Infectious Diseases and Center for Infectious Diseases Research, Houston Methodist Hospital and Houston Methodist Research Institute, Houston, TX, USA (C A Arias MD); Department of Medicine, University of California San Francisco, San Francisco, CA, USA (H F Chambers MD); Division of Infectious Diseases, University of Pittsburgh School of Medicine, Pittsburgh, PA, USA (Y Doi MD); Departments of Microbiology and Infectious Diseases, Fujita Health University School of Medicine, Aichi, Japan (Y Doi); Division of Infectious Diseases, University of North Carolina, Chapel Hill, NC, USA (D van Duin MD); Division of Infectious Diseases, Weill Cornell Medicine, New York, NY, USA (M J Satlin MD)

## Abstract

**Background:**

Carbapenem-resistant *Pseudomonas aeruginosa* (CRPA) is a global threat, but the distribution and clinical significance of carbapenemases are unclear. The aim of this study was to define characteristics and outcomes of CRPA infections and the global frequency and clinical impact of carbapenemases harboured by CRPA.

**Methods:**

We conducted an observational, prospective cohort study of CRPA isolated from bloodstream, respiratory, urine, or wound cultures of patients at 44 hospitals (10 countries) between Dec 1, 2018, and Nov 30, 2019. Clinical data were abstracted from health records and CRPA isolates were whole-genome sequenced. The primary outcome was 30-day mortality from the day the index culture was collected. We compared outcomes of patients with CRPA infections by infection type and across geographic regions and performed an inverse probability weighted analysis to assess the association between carbapenemase production and 30-day mortality.

**Findings:**

We enrolled 972 patients (USA n=527, China n=171, south and central America n=127, Middle East n=91, Australia and Singapore n=56), of whom 581 (60%) had CRPA infections. 30-day mortality differed by infection type (bloodstream 21 [30%] of 69, respiratory 69 [19%] of 358, wound nine [14%] of 66, urine six [7%] of 88; p=0·0012) and geographical region (Middle East 15 [29%] of 52, south and central America 20 [27%] of 73, USA 60 [19%] of 308, Australia and Singapore three [11%] of 28, China seven [6%] of 120; p=0·0002). Prevalence of carbapenemase genes among CRPA isolates also varied by region (south and central America 88 [69%] of 127, Australia and Singapore 32 [57%] of 56, China 54 [32%] of 171, Middle East 27 [30%] of 91, USA ten [2%] of 527; p<0·0001). KPC-2 (n=103 [49%]) and VIM-2 (n=75 [36%]) were the most common carbapenemases in 211 carbapenemase-producing isolates. After excluding USA patients, because few US isolates had carbapenemases, patients with carbapenemase-producing CRPA infections had higher 30-day mortality than those with non-carbapenemase-producing CRPA infections in both unadjusted (26 [22%] of 120 *vs* 19 [12%] of 153; difference 9%, 95% CI 3–16) and adjusted (difference 7%, 95% CI 1–14) analyses.

**Interpretation:**

The emergence of different carbapenemases among CRPA isolates in different geographical regions and the increased mortality associated with carbapenemase-producing CRPA infections highlight the therapeutic challenges posed by these organisms.

## Introduction

*Pseudomonas aeruginosa* is a leading global pathogen.^1^ Infections due to *P aeruginosa* are common, associated with high mortality, and increasingly carbapenem resistant.^2–4^ For this reason, WHO designated carbapenem-resistant *P aeruginosa* (CRPA) as one of three Critical Priority pathogens.^5^ The Antibacterial Resistance Leadership Group therefore set about to characterise the clinical and molecular epidemiology of CRPA.^6^

Non-enzymatic carbapenem resistance mechanisms are common in *P aeruginosa*.^1^ Although the emergence of carbapenemases fuelled the expansion of carbapenem-resistant Enterobacterales,^7^ the extent to which carbapenemases contribute to CRPA globally is unclear. The emergence of carbapenemases in CRPA would have therapeutic implications, because many carbapenemases confer resistance not only to carbapenems, but also to other β-lactam drugs, including some novel β-lactam–β-lactamase inhibitors.^8^ This expanded spectrum of resistance might be associated with worse outcomes among patients with CRPA infections. Furthermore, most rapid diagnostic tests for carbapenem resistance rely on detection of carbapenemase genes,^9^ and thus the utility of these tests to detect CRPA depends on the prevalence and types of carbapenemases harboured by these organisms.

Previous epidemiological investigations of CRPA were geographically limited or lacked detailed clinical data or molecular characterisation of bacteria.^8,10–16^ To address these knowledge gaps, we aimed to identify clinical characteristics of patients with CRPA isolates and outcomes of patients infected with CRPA across geographical regions, characterise the genetic back-grounds and frequency and types of carbapenemases among CRPA isolates across geographical regions, and compare outcomes of patients infected with carbapenemase-producing CRPA with those infected with non-carbapenemase-producing CRPA.

## Methods

### Study design and participants

The prospective observational *Pseudomonas* study (POP) was a cohort study that included 44 hospitals, including 16 in the USA, ten in south and central America (Colombia n=5, Chile n=2, Argentina n=2, Nicaragua n=1), nine in China, five in Australia, two in Singapore, one in Lebanon, and one in Saudi Arabia. Hospitalised patients with CRPA isolated from a bloodstream, respiratory, urinary, or wound culture between Dec 1, 2018, and Nov 30, 2019, and for whom 30-day outcome data were available, were eligible. Only the first eligible CRPA culture episode per patient was included. Patients were initially enrolled on the basis of detection of carbapenem resistance by the local clinical laboratory, but only those whose isolates were meropenem resistant (minimum inhibitory concen tration [MIC] ≥8 μg/mL) on the basis of broth microdilution testing in a central laboratory were included.^17^ Patients whose isolates were not confirmed to be *P aeruginosa* by whole-genome sequencing were excluded. Ethical approval for the study was obtained through institutional review boards of all health systems involved and the requirement to obtain informed consent was waived.

### Procedures

Clinical data were abstracted from electronic health records at study sites and reviewed until 90 days after hospital discharge. Patients were presumed to be alive unless they were known to have died. Infection and colonisation were defined by previously applied criteria,^7^ except for respiratory isolates, where the clinical diagnosis recorded by physicians in the electronic health records was applied with supporting analyses using standardised criteria.^7^ Hospital-acquired infections were defined as those where the first positive culture was collected more than 2 days after hospital admission.

CRPA isolates underwent antimicrobial susceptibility testing at each site’s local clinical microbiology laboratory as per standard of care. Isolates were then sent to a central laboratory (the Antibacterial Resistance Leadership Group Laboratory Center at the Mayo Clinic [Rochester, MN, USA] for isolates not from China and the MDRO Regional Central Laboratory at Huashan Hospital, Fudan University [Shanghai, China] for Chinese isolates) where meropenem susceptibility was assessed by reference broth microdilution.^17^ DNA were extracted using the Wizard Genomic DNA Purification Kit (Promega; Madison, WI, USA) or DNeasy Blood and Tissue Kit (QIAGEN; Venlo, Netherlands). We used Illumina Nextera XT DNA sample preparation kits (Illumina; San Diego, CA, USA) to prepare libraries for sequencing. Isolates underwent next-generation sequencing using an Illumina HiSeq 4000, NextSeq 2000, or MiSeq, as previously described.^18^ We multiplexed and sequenced samples to yield a sequence coverage of around 100x. Paired end-reads were either 150 bp or 300 bp, and the MiSeq Reagent Kit version 3 or HiSeq X Ten Reagent Kit version 2.5 were used. Raw and quality trimmed fastq files were evaluated using Raspberry version 0.3. Sequencing data were quality trimmed and Illumina Nextera indexes removed using Trimmomatic version 0.39. Draft genomes were assembled using SPAdes version 3.13.0. *Pseudomonas* species were determined by fastANI version 1.32, using a 95% cutoff for species identification.^19,20^ Ten genomes were also included that had 94–95% average nucleotide identity with *P aeruginosa* but less than 86% average nucleotide identity with other *Pseudomonas* species. Multilocus sequence typing was analysed using mlst version 2.19.0 and the PubMLST database.^21^ Resistance genes were identified by AMRFinderPlus version 3.10.5 and ARIBA version 2.14.6.^22,23^ Core genome alignment was generated by Snippy version 4.6.0^18^ using the *P aeruginosa* PAO1 genome (accession number NC_002516) as the reference. A maximum likelihood phylogenetic tree was constructed in RAxML version 8.2.4.^24^ *oprD* and its promoter regions were examined by BLASTn from BLAST+ 2.11.0,^25^ and those with a premature stop codon, frameshift, truncation, or promoter region IS insertion were classified as *oprD* mutants.

### Outcomes

The primary outcome was 30-day mortality from the day the index culture was collected. Secondary outcomes were length of hospital stay from the day of the index culture, disposition after hospital discharge, and the desirability of outcome ranking (DOOR) analysis at 30 days.^26^ The DOOR outcome was defined a priori and assessed three undesirable events (absence of clinical response, lack of discharge or hospital readmission, and renal failure or *Clostridioides difficile* infection) and ordered outcomes based on the number of events.^7^ Clinical response was defined as symptomatic response, no additional CRPA therapy after the initial treatment course, and no relapse within 30 days.

### Statistical analysis

We compared characteristics of patients with CRPA isolates and outcomes of patients with CRPA infections between five geographical regions (USA, China, south and central America, the Middle East, and Australia and Singapore). We used the χ2 test, to compare categorical variables, and the Kruskal-Wallis test, to compare continuous variables, in the comparison of characteristics and outcomes of patients with carbapenemase-producing CRPA infections with those with non-carbapenemase-producing CRPA infections. We constructed 95% Wald CIs with pooled variance for differences in 30-day and 90-day mortality. US patients were excluded from this analysis, because few US isolates harboured a carbapenemase. To adjust for confounding in the association between carbapenemases and mortality, we performed an inverse probability weighted analysis with adjustments for geographical region, age-adjusted Charlson Comorbidity Index,^27^ patient location before hospitalisation, immunocompromised status, and anatomical source of infection. We also performed an inverse probability weighted analysis within each geographical region. We visualised 30-day mortality by geographical region and by presence of a carbapenemase with Kaplan-Meier curves with administrative censoring at 30 days. We estimated pairwise DOOR probabilities of a favourable outcome (ie, fewer undesirable events of absence of clinical response, lack of discharge or hospital readmission, and renal failure or *Clostridioides difficile* infection) for a randomly selected infected patient between geographical regions and between patients with carbapenemase-producing and non-carbapenemase-producing CRPA infections. To further assess the association between carbapenemases and mortality, we constructed a multivariate logistic regression model with random effects for study site and a multivariate Cox proportional hazards model, using the same covariates as the inverse probability weighted analysis. p values of 0·05 or less were designated statistically significant and all tests were two-sided. Analyses were performed using SAS version 9.4.

### Role of the funding source

The funder of the study had no role in study design, data collection, data analysis, data interpretation, or writing of the report.

## Results

Of 1443 patients enrolled in POP, 972 (67%) were eligible for this analysis, including 527 (59% of total) in the USA, 171 (18%) in China, 127 (13%) in south and central America, 91 (9%) in the Middle East, and 56 (6%) in Australia and Singapore (appendix p 11). Patients in south and central America (median age 56 years, IQR 32–70) and China (59 years, 46–72) were younger than patients in the USA (63 years, 49–73), Middle East (66 years, 46–74), and Australia and Singapore (68 years, 59–79; p<0·0001; table 1), and had fewer comorbidities (south and central America median Charlson Comorbidity Index 1, IQR 0–2; China 1, 1–2; USA 2, 1–4; Middle East 2, 0–4, Australia and Singapore 3, 1–4; p<0·0001). 581 (60%) patients had CRPA infections and 391 (40%) had CRPA colonisation. 358 (62%) of 581 infections were respiratory, 88 (15%) were urinary, 69 (12%) were bloodstream, and 66 (11%) were wound infections (appendix p 2). Acuity of illness was higher among infected patients in the USA compared with other regions (China median Pitt Bacteraemia Score 2, IQR 0–4; Middle East 2, 1–6; Australia and Singapore 2, 0–4; south and central America 3, 0–6; USA 4, 2–6; p<0·0001).

A carbapenemase gene was detected in 211 (22%) of 972 CRPA isolates, including 186 (19%) with one carbapenemase gene and 25 (3%) with two. *Klebsiella pneumoniae* carbapenemase gene (*bla*_KPC-2_) was most common, present as the only carbapenemase gene in 83 isolates (39% of carbapenemase-producing CRPA isolates) and combined with *bla*_VIM-2_ in 20 (9% of carbapenemase-producing CRPA isolates). *bla*_VIM-2_ was present as the only carbapenemase gene in 52 isolates (25% of carbapenemase-producing CRPA isolates). Other common carbapenemase genes were *bla*_NDM-1_ (n=14, 7% of carbapenemase-producing CRPA isolates), *bla*_IMP-1_ (n=13, 6%), and *bla*_GES-5_ (n=12, 6%). Only one isolate had a class D carbapenemase gene (*bla*_OXA-23_).

**Figure 1:**
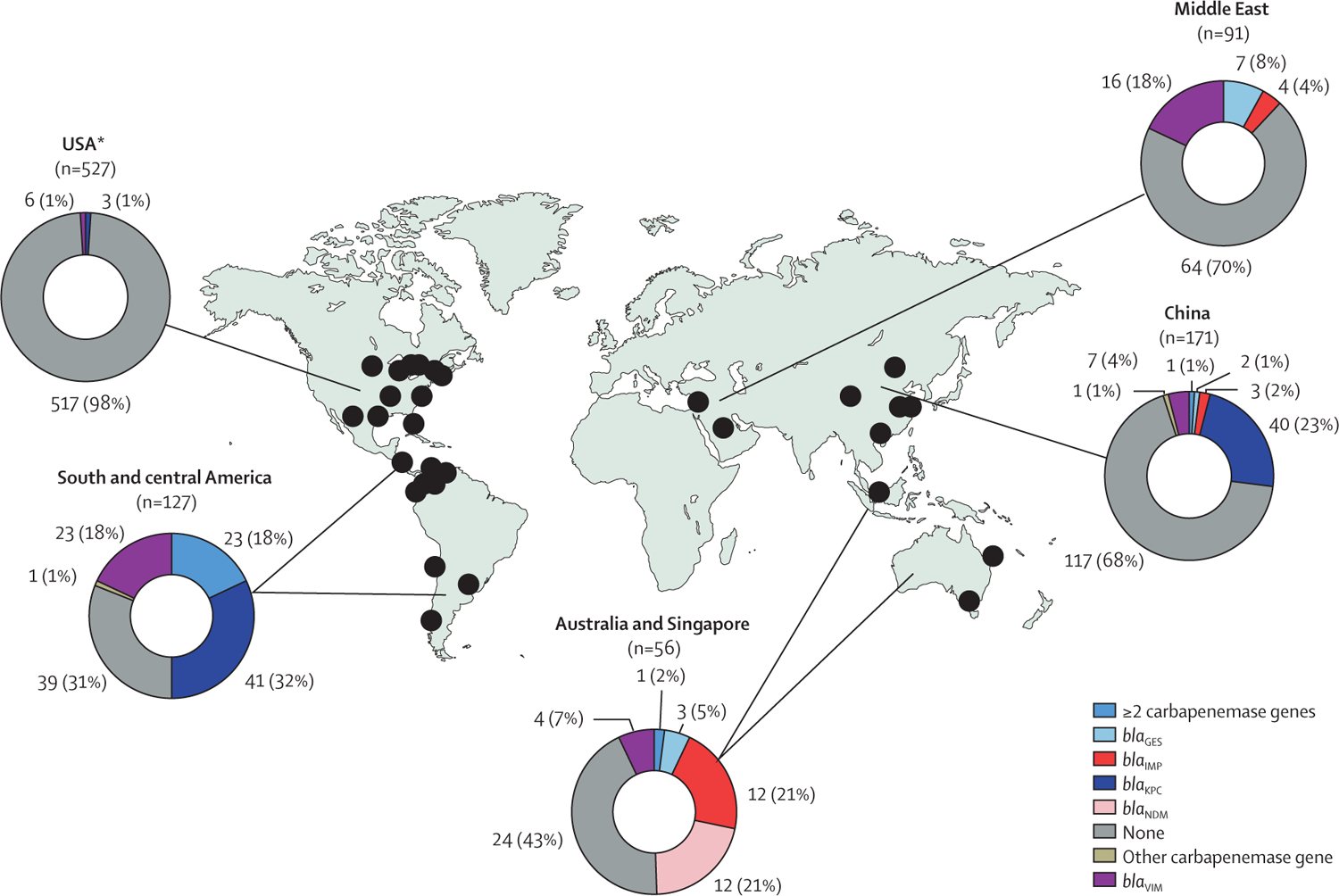
Carbapenemase genes identified in carbapenem-resistant *Pseudomonas aeruginosa* infection and colonisation isolates Isolates carried the followed carbapenemase genes: *bla*_VIM-2_ (n=5), *bla*_KPC-2_ (n=2), *bla*_KPC-3_ (n=1), *bla*_NDM-1_ (n=1), and *bla*_VIM-1_ (n=1) in the USA; *bla*_KPC-2_ (n=40), *bla*_VIM-2_ (n=6), *bla*_GES-5_ (n=2), *bla*_DIM_ (n=1), *bla*_IMP-14_ (n=1), *bla*_IMP-45_ (n=1), *bla*_IMP-54_ (n=1), *bla*_VIM-24_ (n=1), and *bla*_AFM-1_ plus *bla*_IMP-45_ (n=1) in China; *bla*_KPC-2_ (n=41), *bla*_VIM-2_ (n=22), *bla*_KPC-2_ plus *bla*_VIM-2_ (n=20), *bla*_IMP-18_ + *bla*_VIM-2_ (n=3), *bla*_OXA-23_ (n=1), and *bla*_VIM-11_ (n=1) in south and central America; *bla*_VIM-2_ (n=16), *bla*_GES-5_ (n=7), *bla*_IMP-15_ (n=2), *bla*_IMP-1_ (n=1), and *bla*_IMP-13_ (n=1) in the Middle East; and *bla*_IMP-1_ (n=12), *bla*_NDM-1_ (n=12), *bla*_GES-5_ (n=3), *bla*_VIM-2_ (n=3), *bla*_VIM-6_ (n=1), *bla*_IMP-62_ plus *bla*_NDM-1_ (n=1) in Australia and Singapore. *bla_NDM_ was identified in one (<1%) of 527 isolates and is thus not shown in the figure.

88 (69%) of 127 CRPA isolates had a carbapenemase gene in south and central America, 32 (57%) of 56 in Australia and Singapore, 54 (32%) of 171 in China, 27 (30%) of 91 in the Middle East, and 10 (2%) of 527 in the USA (p<0·0001; figure 1). In south and central America, 41 (32%) of 127 isolates harboured *bla*_KPC-2_, 22 (17%) harboured *bla*_VIM-2_, and 20 (16%) harboured both *bla*_KPC-2_ and *bla*_VIM-2_. In China, 40 (23%) of 171 isolates harboured *bla*_KPC-2_ and six (4%) harboured *bla*_VIM-2_. *bla*_VIM-2_ (16 [18%] of 91 isolates) and *bla*_GES-5_ (seven [8%]) were the most common carbapenemase genes in the Middle East. By contrast, *bla*_NDM-1_ (12 [21%] of 56 isolates) and *bla*_IMP-1_ (12 [21%]) were the most common carbapenemase genes in Australia and Singapore. *oprD* mutations were identified in 670 (69%) of 972 isolates and were more frequently present in non-carbapenemase-producing CRPA isolates than in carbapenemase-producing CRPA isolates (546 [72%] of 761 *vs* 124 [59%] of 211; p=0·0003).

Carbapenemase-producing CRPA isolates had higher meropenem MIC values than non-carbapenemase-producing CRPA isolates (appendix p 12). 169 (80%) of 211 carbapenemase-producing CRPA isolates had meropenem MIC values of more than 32 μg/mL, compared with 72 (9%) of 761 non-carbapenemase-producing CRPA isolates (p<0·0001). Carbapenemase-producing CRPA isolates with *oprD* mutations were more likely to have meropenem MIC values of more than 32 μg/mL than those without *oprD* mutations (110 [89%] of 124 *vs* 59 [68%] of 87; p=0·0002). All 13 isolates with *bla*_NDM_, 11 (92%) of 12 isolates with *bla*_GES_, 75 (89%) of 84 isolates with *bla*_KPC_, 16 (84%) of 19 isolates with *bla*_IMP_, and 32 (57%) of 56 isolates with *bla*_VIM_ had meropenem MIC values of more than 32 μg/mL. Local laboratory antimicrobial susceptibility testing indicated that carbapenemase-producing CRPA isolates were less likely to be susceptible to cefepime (14 [7%] of 193 *vs* 289 [42%] of 694), ceftazidime (6 [3%] of 176 *vs* 172 [39%] of 439), piperacillin-tazobactam (eight [5%] of 146 *vs* 252 [36%] of 700), ciprofloxacin (12 [6%] of 202 *vs* 256 [35%] of 730), and amikacin (77 [37%] of 206 *vs* 500 [85%] of 590) than non-carbapenemase-producing CRPA isolates (p<0·0001 for all comparisons; appendix p 3).

**Figure 2:**
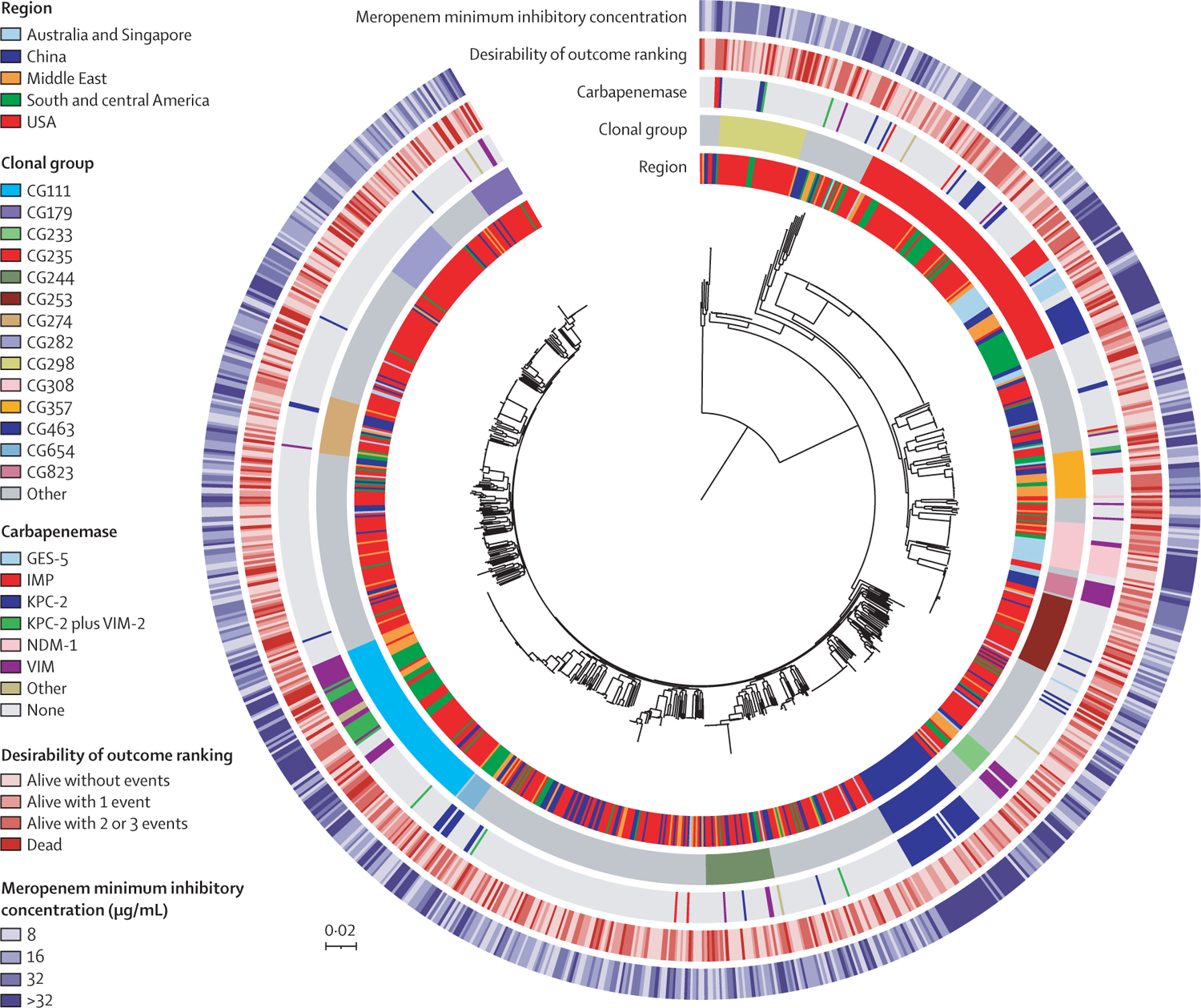
Phylogenetic population structures of carbapenem-resistant *Pseudomonas aeruginosa* isolates

We found diverse genetic lineages among CRPA isolates (figure 2). The most common clonal groups were CG235, representing 117 (12%) of 972 isolates (116 [99%] of 117 were ST235, and one [1%] was ST3746), and CG111, representing 79 (8%) of 972 isolates (69 [87%] of 79 were ST111, nine [11%] were ST966, and one [1%] was other; appendix p 4). No other clonal group represented more than 4% of isolates. CG235 was the most common clonal group in south and central America (34 [27%] of 127 isolates), Australia and Singapore (13 [23%] of 56), the Middle East (14 [15%] of 91), and the USA (50 [9%] of 527). CG111 was the next most common clonal group in south and central America (26 [20%] of 127) and the Middle East (12 [13%] of 91), and 37 (97%) of 38 CG111 isolates in these regions harboured *bla*_VIM-2_. 14 (54%) of 26 CG111 isolates in south and central America harboured both *bla*_KPC-2_ and *bla*_VIM-2_. In China, CG463 (all ST463) was most common (34 [20%] of 171 isolates), and 31 (91%) of 34 CG463 isolates harbored *bla*_KPC-2_. CG463 was not identified in any other region. CG308 (20 [95%] of 21 were ST308, and one [5%] was ST2126) was most common in Australia and Singapore, where all 12 CG308 isolates harboured *bla*_NDM-1_.

**Figure 3:**
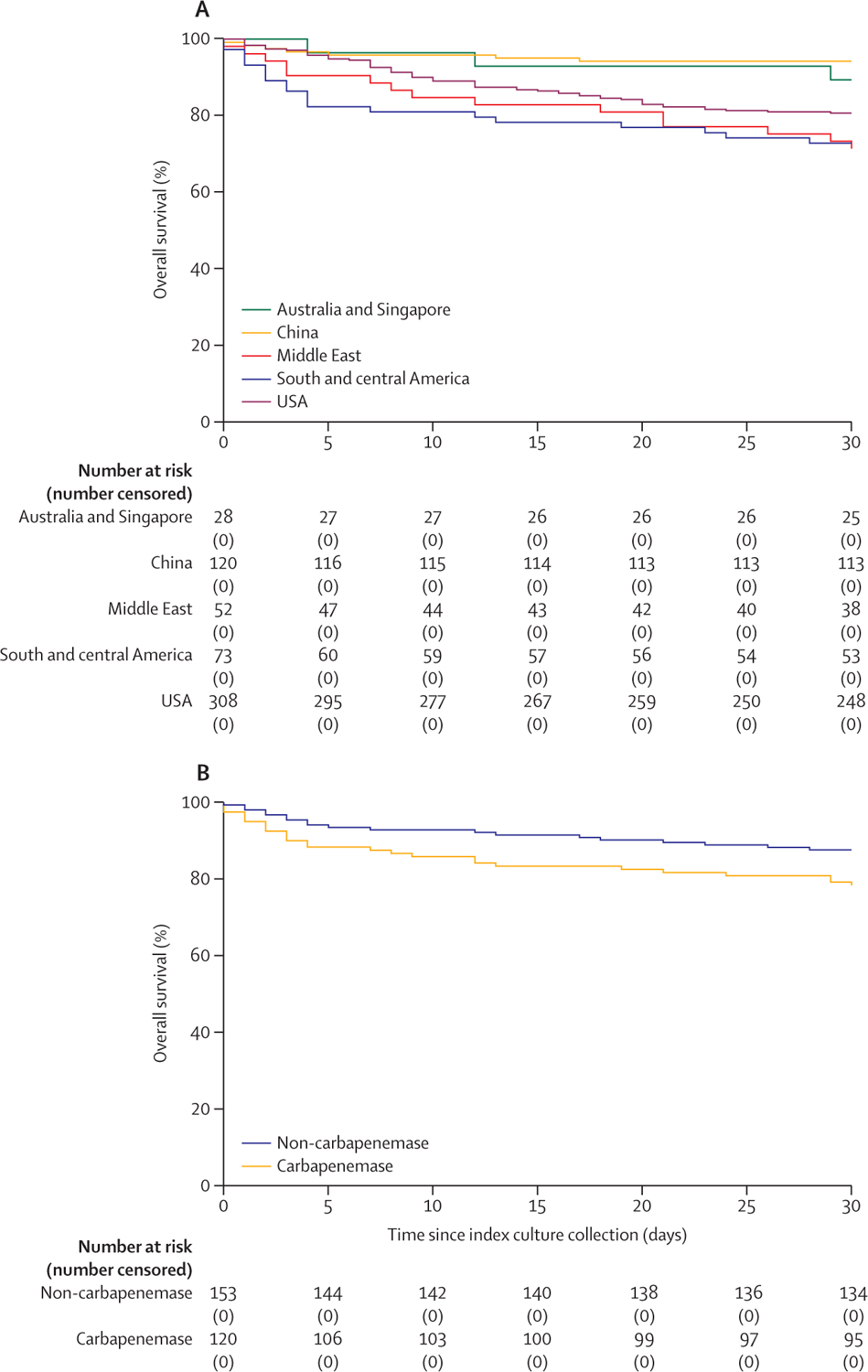
30-day overall survival after carbapenem-resistant *Pseudomonas aeruginosa* infection (A) 30-day overall survival by region. (B) 30-day overall survival by presence of carbapenemase for infections outside the USA.

105 (18%, 95% CI 15–21) of 581 patients infected with CRPA died within 30 days and 148 (25%, 22–29) died within 90 days (table 2). 21 (30%, 20–41) of 69 patients with bloodstream infections died within 30 days, 69 (19%, 15–23) of 358 patients with respiratory infections, nine (14%, 5–22) of 66 patients with wound infections, and six (7%, 2–12) of 88 patients with urinary infections (p=0·0012; appendix p 13). 223 (38%, 95% CI 34–42) of 581 infected patients were discharged to their home. 15 (29%, 17–41%) of 52 patients died within 30 days in the Middle East, 20 (27%, 17–38) of 73 in south and central America, 60 (19%, 15–24) of 308 in the USA, three (11%, 0–22) of 28 in Australia and Singapore, and seven (6%, 2–10) of 120 in China (p=0·0002; figure 3A; table 2). Similar mortality was observed when applying a standardised definition of respiratory tract infection instead of a physician-adjudicated definition (appendix p 5). Probabilities of favourable DOOR outcomes were greater in China than in other geographical regions (table 2; appendix p 14).

120 (44%) of 273 patients with CRPA infections outside of the USA were infected by carbapenemase-producing CRPA. Patients with carbapenemase-producing CRPA infections were more likely to be in south and central America (53 [44%] of 120 with carbapenemase-producing CRPA *vs* 20 [13%] of 153 with non-carbapenemase-producing CRPA), immunocom promised (23 [19%] *vs* 11 [7%]), and have a bloodstream (25 [21%] *vs* 15 [10%]) or urinary infection (36 [30%] *vs* 5 [3%]) than patients with non-carbapenemase-producing CRPA infections, and were less likely to be in China (30 [25%] *vs* 90 [59%]) and have a respiratory (44 [37%] *vs* 120 [78%]) or polymicrobial infection (33 [28%] *vs* 66 [43%]; appendix p 6). Mortality was higher in patients with carbapenemase-producing CRPA infections compared with non-carbapenemase-producing CRPA infections at 30 days (26 [22%] of 120 *vs* 19 [12%] of 153; unadjusted difference 9%, 95% CI 3–16; figure 3B) and at 90 days (33 [28%] of 120 *vs* 28 [18%] of 153; unadjusted difference 9%, 2–16). Carbapenemase-producing CRPA infections were also associated with increased mortality compared with non-carbapenemase-producing CRPA in an inverse probability weighted analysis (30-day difference 8%, 1–14; 90-day difference 8%, 0–15). Increased 30-day mortality after carbapenemase-producing CRPA infection was also observed within each geographical region, except for south and central America (appendix p 7), and when performing an analysis that applied a standardised definition of CRPA pneumonia (appendix p 8). The multivariate logistic regression (adjusted odds ratio 2∙09, 95% CI 0∙93–4∙70) and Cox proportional hazards models (adjusted hazard ratio 1∙41, 95% CI 0∙71–2∙80) also suggested increased 30-day mortality with carbapenemase production (appendix pp 9–10). DOOR outcomes, clinical response rates, and hospital length of stay were not significantly different between carbapenemase-producing CRPA and non-carbapenemase-producing CRPA infections (appendix p 6).

## Discussion

In this international, prospective cohort study, we found differences in the prevalence and types of carbapenemases harboured by CRPA across geographical regions. Although only 2% of CRPA isolates from the USA had a carbapenemase, 30–69% of CRPA isolates in other regions had a carbapenemase, with the highest frequency in south and central America. KPC-2 was the most common carbapenemase globally, followed by VIM-2, IMP-1, NDM-1, and GES-5, but distinct carbapenemases predominated in different regions. Carbapenemase-producing CRPA isolates were more likely to have high-level meropenem resistance and resistance to other anti-pseudomonal drugs than non-carbapenemase-producing CRPA isolates. Moreover, carbapenemase-producing CRPA infections were associated with increased mortality compared with non-carbapenemase-producing CRPA infections (22% *vs* 12%), with this difference persisting after adjusting for age, geographical region, comorbidities, patient location before admission, immunocompromised status, and source of infection.

This work was conducted through the Multi-drug Resistant Organism Network. It was a multinational study aimed at increasing understanding of the clinical and molecular epidemiology of carbapenem-resistant Gram-negative pathogens, and adds to previous analyses of carbapenem-resistant Enterobacterales and *Acinetobacter baumannii* from the Multi-drug Resistant Organism Network.^7,18,28^ Most previous studies of carbapenemases in *P aeruginosa* analysed isolates within a single country or region.^8,10–15^ An exception is an analysis of CRPA isolates from 12 countries by Gill and colleagues.^16^ They found that VIM and GES were the most common carbapenemases, which differs from our study, in which KPC was most common. We believe that differences in geographical regions between studies might explain these disparate findings. *bla*_KPC_ has driven the global proliferation of carbapenem resistance in Enterobacterales,^7^ so the emergence of KPC in *P aeruginosa* poses a major public health threat, particularly because KPC-producing organisms are resistant to the anti-pseudomonal drug ceftolozane-tazobactam.^29^ Moreover, we found that *P aeruginosa* harbouring both *bla*_KPC-2_ and *bla*_VIM-2_ has emerged in south and central America. The presence of both enzymes reduces treatment options because avibactam and relebactam do not inhibit VIM carbapenemases, and thus these organisms are also resistant to ceftazidime–avibactam and imipenem-relebactam.^29,30^ We hypothesise that the expanded resistance of carbapenemase-producing CRPA might have contributed to the increased mortality observed among patients infected with carbapenemase-producing CRPA compared with non-carbapenemase-producing CRPA.

Our finding that carbapenem resistance in *P aeruginosa* is rarely due to carbapenemases in the USA corroborates work from the Centers for Disease Control and Prevention’s Antibacterial Resistance Laboratory Network. They sampled CRPA isolates submitted to USA public health laboratories, used targeted PCR or phenotypic methods to identify carbapenemases, and found that 3% of CRPA isolates possessed a carbapenemase.^11^ We believe that identifying a similarly low prevalence of carbapenemases in our cohort using whole-genome sequencing strengthens the conclusion that carbapenemase-producing CRPA are rare in the USA. However, surveillance is needed to detect the emergence of carbapenemase-producing CRPA in the USA, given their emergence in other regions.

We identified substantial differences in outcomes of patients with CRPA infection by geographical region. 30-day mortality was 6% in China and 11% in Australia and Singapore, but it was 19% in the USA, 27% in south and central America, and 29% in the Middle East. The reasons for these mortality differences are unclear, but mortality was also lower in China in an international study of infections caused by carbapenem-resistant *Klebsiella pneumoniae*.^18^ Patients from China had lower Pitt Bacteraemia Scores, indicating a lower severity of acute illness, and were less likely to be immunocompromised than patients from other regions. It is possible that differences in characteristics of health-care systems and supportive care contributed to these mortality differences. Ceftolozane-tazobactam and ceftazidime-avibactam were not available or not widely used during the study in south-central American countries and China, but one or both drugs were frequently used in other regions. The unavailability of these drugs in south and central America, combined with the high prevalence of carbapenemase-producing CRPA that are resistant to traditional anti-pseudomonal drugs, might have contributed to the high mortality observed in this region. These geographical differences in outcomes have implications for clinical trials of treatments for CRPA infections. A clinical trial of ceftolozane-tazobactam might show efficacy in the USA, where carbapenemase-producing CRPA is rare, but not in south and central America, where KPC-producing and VIM-producing CRPA are common.

The genetic heterogeneity of CRPA isolates in this study contrasts with the clonal dominance of ST258 and ST11 in carbapenem-resistant *K pneumoniae* or ST2 in carbapenem-resistant *A baumannii*.^18,28^ Although CG235 was most common, it represented only 12% of CRPA isolates in this study. Different clones within CG235 have acquired different carbapenemase genes in different geographical regions. For example, although CG235 did not harbour carbapenemases in the USA, a CG235 clone acquired *bla*_KPC-2_ in south and central America, another CG235 clone acquired *bla*_GES-5_ in the Middle East, and another acquired *bla*_IMP-1_ in Australia and Singapore. Although CG111 was observed in most regions, only CG111 strains in south and central America acquired both *bla*_KPC-2_ and *bla*_VIM-2_. Additionally, certain clonal groups that acquired carbapenemase genes have emerged within specific regions. For example, CG463 was only identified in China and almost all isolates possessed *bla*_KPC-2_. CG308 was mostly identified in Australia and Singapore, where it had acquired *bla*_NDM-1_. Although these emerging high-risk carbapenemase-producing CRPA might currently be geographically limited, vigilance is warranted to detect their emergence in new locations.

This study has limitations. Although it included hospitals from four continents, it did not include sites from Europe or Africa. Furthermore, participating hospitals might not be completely representative of their geographical region. The clinical data only included data that were available through each hospital’s electronic health records. Thus, it is possible that differences in documentation contributed to geographical variation in patient characteristics. This approach was pursued to obtain a waiver of informed consent, which permitted consecutive enrolment of patients with CRPA infections at study hospitals without selection bias. Furthermore, our primary outcome of 30-day mortality does not rely on extensive documentation in electronic health records. The in-vitro activity of non-carbapenem antibiotics against CRPA isolates was assessed using antimicrobial susceptibility testing results from local laboratories, not central laboratories. Although this testing was not standardised, it represents real-world data that were available to providers. It is a strength that whole-genome sequencing was performed on all CRPA isolates, but even whole-genome sequencing is insufficient to identify all mechanisms of carbapenem resistance in *P aeruginosa*, such as overexpression of efflux pumps and chromosomal β-lactamases. We encourage additional studies of CRPA that assess gene expression to garner additional insights. Although we adjusted for potential confounders using inverse probability weighting, we might have been unable to adjust for all variables that might confound the association between carbapenemase-producing CRPA and mortality. Furthermore, this association might not apply to US patients, because they were excluded from this analysis owing to a low prevalence of carbapenemases. We encourage additional investigations to confirm our findings in other cohorts. Finally, we evaluated CRPA isolated from the four most common anatomical sites, but our results might not apply to other anatomical sites.

In summary, this multinational study identified differences in the clinical characteristics and outcomes of patients infected with CRPA across geographical regions. Carbapenemases were rare in CRPA isolates in the USA but common in isolates in other regions, particularly KPC-2 and VIM-2. Carbapenemase-producing CRPA isolates exhibited higher degrees of meropenem resistance than non-carbapenemase-producing CRPA isolates and were more frequently resistant to other anti-pseudomonal drugs. Moreover, patients with carbapenemase-producing CRPA infection had a higher 30-day mortality rate, even after adjustment for confounders. These findings highlight that different strategies are needed to combat CRPA in different parts of the world.

## Supplementary Material

1

## Figures and Tables

**Table 1:** Patient characteristics

	Total (n=972)	USA (n=527)	China (n=171)	South and central America (n=127)	Middle East (n=91)	Australia and Singapore (n=56)	p value*
Demographics							
Age, years	62 (47–73)	63 (49–73)	59 (46–72)	56 (32–70)	66 (46–74)	68 (59–79)	<0·0001
Sex	··	··	··	··	··	··	0·052
Female	351 (36%)	206 (39%)	48 (28%)	42 (33%)	38 (42%)	17 (30%)	··
Male	621 (64%)	321 (61%)	123 (72%)	85 (67%)	53 (58%)	39 (70%)	··
Comorbidities							
Charlson Comorbidity Index	2 (1–4)	2 (1–4)	1 (1–2)	1 (0–2)	2 (0–4)	3 (1–4)	<0·0001
Diabetes	330 (34%)	193 (37%)	31 (18%)	37 (29%)	39 (43%)	30 (54%)	<0·0001
Heart disease	271 (28%)	192 (36%)	27 (16%)	11 (9%)	28 (31%)	13 (23%)	<0·0001
Cerebrovascular disease	200 (21%)	100 (19%)	63 (37%)	9 (7%)	22 (24%)	6 (11%)	<0·0001
Chronic kidney disease	126 (13%)	90 (17%)	5 (3%)	11 (9%)	15 (16%)	5 (9%)	<0·0001
Chronic obstructive pulmonary disease	154 (16%)	119 (23%)	11 (6%)	8 (6%)	10 (11%)	6 (11%)	<0·0001
History of malignancy	180 (19%)	91 (17%)	27 (16%)	28 (22%)	21 (23%)	13 (23%)	0·34
Immunocompromised	123 (13%)	77 (15%)	7 (4%)	24 (19%)	11 (12%)	4 (7%)	0·0007
Origin of patient	··	··	··	··	··	··	<0·0001
Home	533 (55%)	261 (50%)	83 (49%)	79 (62%)	70 (77%)	40 (71%)	··
Long-term care facility	171 (18%)	150 (28%)	7 (4%)	2 (2%)	2 (2%)	10 (18%)	··
Hospital transfer	261 (27%)	115 (22%)	81 (47%)	44 (35%)	16 (18%)	5 (9%)	··
Foreign country	6 (1%)	1 (<1%)	0	1 (1%)	3 (3%)	1 (2%)	··
Hospice	1 (<1%)	0	0	1 (1%)	0	0	··
Previous intensive care unit admission	549 (56%)	296 (56%)	92 (54%)	73 (57%)	68 (75%)	20 (36%)	0·0001
Patient location at time of first positive culture	··	··	··	··	··	··	<0·0001
Emergency department	119 (12%)	93 (18%)	5 (3%)	20 (16%)	0	1 (2%)	··
Hospital ward	418 (43%)	185 (35%)	97 (57%)	43 (34%)	47 (52%)	46 (82%)	··
Intensive care unit	402 (41%)	233 (44%)	66 (39%)	53 (42%)	41 (45%)	9 (16%)	··
Other	33 (3%)	16 (3%)	3 (2%)	11 (9%)	3 (3%)	0	··
Days from admission to culture	8 (1–27)	4 (0–21)	10 (2–25)	20 (2–43)	22 (9–65)	13 (1–25)	<0·0001
Hospital-acquired	609 (63%)	281 (53%)	128 (75%)	90 (71%)	74 (81%)	36 (64%)	<0·0001
Infection or colonisation by source	··	··	··	··	··	··	<0·0001
Blood (infection only)	69 (7%)	29 (6%)	10 (6%)	15 (12%)	12 (13%)	3 (5%)	··
Respiratory	523 (54%)	283 (54%)	126 (74%)	41 (32%)	62 (68%)	11 (20%)	··
Infection	358 (37%)	194 (37%)	94 (55%)	30 (24%)	31 (34%)	9 (16%)	··
Colonisation	165 (17%)	89 (17%)	32 (19%)	11 (9%)	31 (34%)	2 (4%)	··
Urinary	214 (22%)	120 (23%)	23 (13%)	42 (33%)	8 (9%)	21 (38%)	··
Infection	88 (9%)	47 (9%)	11 (6%)	19 (15%)	6 (7%)	5 (9%)	··
Colonisation	126 (13%)	73 (14%)	12 (7%)	23 (18%)	2 (2%)	16 (29%)	··
Wound	166 (17%)	95 (18%)	12 (7%)	29 (23%)	9 (10%)	21 (38%)	··
Infection	66 (7%)	38 (7%)	5 (3%)	9 (7%)	3 (3%)	11 (20%)	··
Colonisation	100 (10%)	57 (11%)	7 (4%)	20 (16%)	6 (7%)	10 (18%)	··
Pitt Bacteraemia Score	3 (1–6)	3 (2–6)	2 (0–4)	2 (0–6)	3 (1–6)	2 (0–4)	<0·0001
Polymicrobial	345 (35%)	191 (36%)	78 (46%)	31 (24%)	19 (21%)	26 (46%)	<0.0001

Data are n (%) or median (IQR).

* χ^2^ test was used for categorical variables, and Kruskal-Wallis test was used for continuous variables.

**Table 2:** Patient outcomes

	Total (n=581)	USA (n=308)	China (n=120)	South and central America (n=73)	Middle East (n=52)	Australia and Singapore (n=28)	p value*
Mortality†							
30-day (primary outcome)	105 (18%)	60 (19%)	7 (6%)	20 (27%)	15 (29%)	3 (11%)	0·0002
90-day	148 (25%)	87 (28%)	10 (8%)	23 (32%)	21 (40%)	7 (25%)	<0·0001
Length of hospital stay from infection onset, days	13 (6–30)	10 (5–20)	15 (7–30)	18 (7–41)	28 (11–59)	39 (19–72)	<0·0001
Desirability of outcomes ranking outcome at 30 days‡							
Alive without events	211 (36%)	120 (39%)	51 (43%)	22 (30%)	12 (23%)	6 (21%)	··
Alive with 1 event	123 (21%)	73 (24%)	36 (30%)	9 (12%)	2 (4%)	3 (11%)	··
Alive with 2 or 3 events	142 (24%)	55 (18%)	26 (22%)	22 (30%)	23 (44%)	16 (57%)	··
Death	105 (18%)	60 (19%)	7 (6%)	20 (27%)	15 (29%)	3 (11%)	··
Disposition after discharge							<0·0001
Home	223 (38%)	94 (31%)	47 (39%)	42 (58%)	27 (52%)	13 (46%)	··
Long-term care facility	145 (25%)	129 (42%)	4 (3%)	5 (7%)	1 (2%)	6 (18%)	··
Transfer to another hospital or to a foreign country	57 (10%)	5 (2%)	48 (40%)	2 (3%)	1 (2%)	1 (4%)	··
Hospice	27 (5%)	15 (5%)	11 (9%)	1 (1%)	0	0	··
Death	121 (21%)	63 (20%)	10 (8%)	22 (30%)	20 (38%)	6 (21%)	··
Remained in the hospital	8 (1%)	2 (1%)	0	1 (1%)	3 (6%)	2 (7%)	··
Clinical response	283 (49%)	175 (57%)	56 (47%)	29 (40%)	14 (27%)	9 (32%)	<0·0001

Data are n (%) or median (IQR).

* χ^2^ test was used for categorical variables, and Kruskal-Wallis test was used for continuous variables.

† Patients who were discharged to hospice were not considered to have died.

‡ The three adverse events assessed by desirability of outcomes ranking were lack of clinical response, lack of discharge within 30 days or readmission within 30 days, and incident renal failure or *Clostridioides difficile* infection. The unadjusted probability estimates for a favourable outcome (ie, clinical response, discharge or hospital readmission, and absence of renal failure or *Clostridioides difficile* infection) using China as the reference region were 44% (95% CI 39–50) for the USA, 35% (27–43) for south and central America, 28% (20–37) for the Middle East, and 31% (21–42) for Australia and Singapore (appendix p 5).

## Data Availability

Individual deidentified participant data (and supporting documentation, data dictionaries, and protocol) that underlie the results in this Article can be made available to investigators following submission of a plan for data use, approval by the Antibacterial Resistance Leadership Group or designated entity, and execution of required institutional agreements. Provision might be contingent upon the availability of funding for data preparation and deidentification. More information can be found on the Antibacterial Resistance Leadership Group website. Sequences will be publicly available through the National Center for Biotechnology Information (accession number PRJNA824880).

## Research in context

### Evidence before this study

We searched PubMed without language restrictions for articles published before May 17, 2022, using the terms “carbapenem resistant”, “*Pseudomonas aeruginosa*”, and “carbapenemase”. The results of this search primarily identified studies of carbapenem-resistant *Pseudomonas aeruginosa* (CRPA) epidemiology that were conducted in individual centres or individual countries. For example, studies conducted by the Centers for Disease Control and Prevention found that 2–3% of CRPA isolates submitted to public health laboratories in the USA possessed a carbapenemase. We identified one study that characterised CRPA isolates from 14 countries in the Asia-Pacific region and found that VIM, NDM, VEB, and IMP were the most common carbapenemases harboured by these organisms.

A second international study analysed CRPA isolates from17 medical centres in 12 countries in Europe, the Middle East, the USA, South America, and Africa, and found that 33% of isolates possessed carbapenemases, of which VIM and GES were most common. Both studies found that carbapenemase-producing CRPA isolates were less likely to test susceptible to other anti-pseudomonal drugs than non-carbapenemase-producing CRPA isolates. Neither of these studies used whole-genome sequencing to provide a comprehensive evaluation of carbapenemase genes and detailed clinical data to assess patient characteristics and outcomes were not available. We did not identify any studies that compared outcomes of patients infected with CRPA across different geographical regions or compared outcomes of patients infected with carbapenemase-producing CRPA with those infected with non-carbapenemase-producing CRPA.

### Added value of this study

We used a uniform protocol to characterise the clinical and bacterial characteristics of all CRPA isolates from 972 patients hospitalised over a 1-year period at 44 medical centres in10 countries. We made several observations not made in previous studies. First, 30-day mortality after CRPA infection was highest in bloodstream infections (30%), followed by infections of the respiratory tract (19%), wounds (14%), and urinary tract (7%). Second, 30-day mortality varied across geographical regions, with the highest mortality in the Middle East (29%) and south and central America (27%), and the lowest in China (6%). Third, 30-day mortality was higher in patients with carbapenemase-producing CRPA infections compared with non-carbapenemase-producing CRPA infections (22% *vs* 12%) and this mortality difference persisted even after adjusting for age, comorbidities, geographical region, patient location before hospitalisation, immunocompromised status, and anatomical source of infection. These new findings highlight the differences in the role of carbapenemases and in clinical outcomes in CRPA infections across different geographical regions.

### Implications of all the available evidence

The differential emergence of carbapenemases among CRPA isolates across geographical regions has diagnostic and therapeutic implications. Given that most rapid diagnostic tests for carbapenem resistance rely on detecting carbapenemase genes, the yield of these assays to detect CRPA might be lower in the USA than in regions with a high prevalence of carbapenemase-producing CRPA. Furthermore, the emergence of serine-carbapenemases and metallo-carbapenemases in multiple geographical regions reduces the clinical utility of new β-lactam–β-lactamase inhibitors for CRPA infections in these areas, because these new drugs might not be effective against organisms with these enzymes. These findings have implications for the design of clinical trials of new antibacterial drugs for CRPA infections.

## MDRO Network Investigators (listed by centre alphabetically)

American University of Beirut Medical Center, Beirut, Lebanon: Souha S Kanj; Beijing Ditan Hospital, Capital Medical University, Beijing, China: Fujie Zhang; Boston University, Boston, MA, USA: Judith J Lok; Case Western, Cleveland, OH, USA: Robert A Salata; Centro de Educación Médica e Investigaciones Clínicas, Buenos Aires, Argentina: Martin Stryjewski, Valentina Di Castelnuovo; Centro Medico Imbanaco, Cali, Colombia: Jose Millan Oñate Gutierrez; Cleveland Clinic, Cleveland, OH, USA: Eric Cober, Susan Richter; Duke University, Durham, NC, USA: Deverick J Anderson, Beth Evans, Carol Hill, Heather R Cross, Keri Baum, Rebekka Arias, Vance G Fowler Jr; ESE Hospital Universitario, San Jorge de Pereira, Pereira, Colombia: Karen Ordoñez; Emory University, Atlanta, GA, USA: Jesse T Jacob; Guangzhou Eighth People’s Hospital, Guangzhou, China: Linghua Li; Hackensack Meridian Health, Nutley, NJ, USA: Barry N Kreiswirth, Claudia Manca, Liang Chen, Samit Desai; Henry Ford Hospital, Detroit, MI, USA: Erica Herc; Hospital “Cosme Argerich” de Buenos Aires, Buenos Aires, Argentina: Ezequiel Cordova; Hospital de Puerto Montt, Puerto Montt, Chile: Maria Rioseco; Hospital Escuela Oscar Danilo Rosales Arguello, Leon, Nicaragua: Samuel Vichez; Hospital Italiano de Buenos Aires, Buenos Aires, Argentina: Marisa L Sanchez; Hospital San Ignacio, Bogotá, Colombia: Sandra Valderrama; Hospital Universitario Erasmo Meoz ESE, Cúcuta, Colombia: Jairo Figueroa; Houston Methodist, Houston, TX, USA: Cesar A Arias, An Q Dinh, Diane Panesso, Kirsten Rydell, Truc T Tran; Huashan Hospital, Fudan University, Shanghai, China: Fupin Hu, Jiachun Su, Jianping Jiang, Minggui Wang, Xiaogang Xu, Yang; Instituto de Ciencias e Innovación en Medicina, Clinica Alemana, Universidad del Desarrollo, Santiago, Chile: Jose M Munita, Maria Spencer; King Abdulaziz Medical City, Riyadh, Saudi Arabia: Thamer Alenazi; Louis Stokes Cleveland Department of Veterans Affairs Medical Center, Cleveland, OH, USA: Robert A Bonomo, Steven H Marshall, Susan D Rudin; Mayo Clinic, Rochester, MN, USA: Charles Huskins, Kerry Greenwood-Quaintance, Robin Patel, Suzannah Schmidt-Malan; Medical College of Wisconsin, Milwaukee, WI, USA: Sara Revolinski; MedStar Washington Hospital Center, Washington, DC, USA: Glenn Wortmann; MetroHealth Medical Center, Cleveland, OH, USA: Robert C Kalayjian; Montefiore Medical Center, Albert Einstein College of Medicine, New York, NY, USA: Gregory Weston; Montefiore Medical Center, Moses Campus, Bronx, NY, USA: Belinda Ostrowsky; Mount Sinai, New York, NY, USA: Gopi Patel; New York University Langone Medical Center, NY, USA: Daniel Eiras; North Shore University Hospital, Manhasset, NY, USA: Angela Kim; Ochsner Clinic Foundation, New Orleans, LA, USA: Julia Garcia-Diaz; Organizacion Clinica General del Norte, Barranquilla, Colombia: Soraya Salcedo; OSF Saint Francis Medical Center, Peoria, IL, USA: John J Farrell; Peking Union Medical College Hospital, Beijing, China: Fujie Zhang, Zhengyin Liu; Princess Alexandra Hospital, Brisbane, Queensland, Australia: Andrew Henderson; Royal Brisbane and Women’s Hospital, Brisbane, Queensland, Australia: David L Paterson; Ruijin Hospital, Shanghai, China: Qing Xie; Rutgers University, New Brunswick, NJ, USA: Keith S Kaye; Shulan Hangzhou Hospital, Shulan Health, Hangzhou, China: Hainv Gao; Sir Run Shaw Hospital, Zhejiang University School of Medicine, Hangzhou, China: Yunsong Yu; St Vincent’s Hospital, Melbourne, Victoria, Australia: Mary Waters; Stony Brook University, Stony Brook, NY, USA: Bettina C Fries; SUNY Downstate Medical Center, New York, NY, USA: Brandon Eilertson; Tan Tock Seng Hospital, Singapore: Kalisvar Marimuthu, Oon Tek Ng, Partha Pratim De; National University of Singapore, Singapore: Kean Lee Chew, Nares Smitasin, Paul Ananth Tambyah; Temple University Hospital, Philadelphia, PA, USA: Jason C Gallagher; The Alfred Hospital, Melbourne, Victoria, Australia: Anton Peleg; The Austin Hospital, Heidelberg West, Victoria, Australia: Marcel Leroi; The First Affiliated Hospital of Medical School of Zhejiang University, Hangzhou, China: Lanjuan Li; The George Washington University, Washington, DC, USA: Lauren Komarow, Lizhao Ge, Scott Evans; The University of Alabama, Birmingham, AL, USA: Todd McCarty; The University of California San Francisco, CA, USA: Henry F Chambers; The University of California, Los Angeles, CA, USA: Omai B Garner; The University of Miami Miller School of Medicine and Jackson Health System, Miami, FL, USA: Lilian M Abbo; The University of North Carolina, Chapel Hill, NC, USA: David van Duin; The University of Pennsylvania Health System, Philadelphia, PA, USA: Ebbing Lautenbach, Jennifer H Han; The University of Pittsburgh School of Medicine, Pittsburgh, PA, USA: Yohei Doi; The University of Southern California, Los Angeles, CA, USA: Darren Wong; The University of Texas Health Science Center at Houston, Houston, TX, USA: Blake Hanson; Universidad El Bosque, Bogotá, Colombia: Jinnethe Reyes, Maria Virginia Villegas Botero, Lorena Diaz; University Hospitals Cleveland Medical Center, Cleveland, OH, USA: Federico Perez; Vanderbilt University Medical Center, Nashville, TN, USA: Ritu Banerjee; Wayne State University, Detroit, MI, USA: Sorabh Dhar; Weill Cornell Medicine, NewYork-Presbyterian Hospital, New York, NY, USA: Michael J Satlin, Lars F Westblade; West China Hospital of Sichuan University, Chengdu, China: Zhiyong Zong.
