# Supplementary material for: Global epidemiology and clinical outcomes of carbapenem-resistant Pseudomonas aeruginosa and associated carbapenemases (POP): a prospective cohort study
Source: Lancet Microbe. Author manuscript; Available in PMC 2023 Mar 15. (PMC10016089; doi:10.1016/S2666-5247(22)00329-9)

THE LANCET
Microbe

Supplementary appendix

This appendix formed part of the original submission and has been peer reviewed. We post it as supplied by the authors.

Supplement to: Reyes J, Komarow L, Chen L, et al. Global epidemiology and clinical outcomes of carbapenem-resistant *Pseudomonas aeruginosa* and associated carbapenemases (POP): a prospective cohort study. *Lancet Microbe* 2023; published online Feb 9. https://doi.org/10.1016/S2666-5247(22)00329-9.

SUPPLEMENTARY MATERIAL

Table of Contents **Page**

Table of Contents page	1
Supplemental Table 1. Characteristics of patients infected with CRPA by region.	2
Supplemental Table 2. Antimicrobial susceptibilities of carbapenemase-producing vs. non-carbapenemase-producing CRPA isolates based on local laboratory testing.	3
Supplemental Table 3. CRPA Clonal Group by geographic region and associations with carbapenemases and infection (vs. colonization).	4
Supplemental Table 4. Outcomes of patients infected with CRPA by region using a standardized definition of pneumonia instead of a physician-adjudicated assessment.	5
Supplemental Table 5. Characteristics and outcomes of patients with carbapenemase-producing vs. non-carbapenemase-producing CRPA infections outside of the USA.	6
Supplemental Table 6. Unadjusted and IPW-adjusted 30-day mortality (%) among patients infected with CP-CRPA vs. non-CP-CRPA stratified by geographic regions (excluding USA patients).	7
Supplemental Table 7. Unadjusted and IPW-adjusted 30-day mortality (%) among patients infected with CP-CRPA vs. non-CP-CRPA using a standardized definition of pneumonia instead of a physician-adjudicated assessment.	8
Supplemental Table 8. Multivariate logistic regression model of baseline covariates and their associations with 30-day mortality after CRPA infection (excluding USA patients, with a random effect for geographic region).	9
Supplemental Table 9. Multivariate Cox proportional hazards model of baseline covariates and their associations with 30-day mortality after CRPA infection (excluding USA patients).	10
Supplemental Figure 1. Flow diagram of patients included in the study.	11
Supplemental Figure 2. Meropenem minimum inhibitory concentration (MIC) distributions of CP-CRPA and non-CP-CRPA.	12
Supplemental Figure 3. DOOR outcomes stratified by culture anatomical source and infection vs. colonization.	13
Supplemental Figure 4. DOOR outcomes stratified by geographical region.	14

Supplemental Table 1. Characteristics of patients infected with CRPA by region.

Characteristics	USA (n=308)	China (n=120)	South-Central America (n=73)	Middle East (n=52)	Australia-Singapore (n=28)	Total (n=581)	*P*[1]
Demographics							
Age	62 (49-72)	59 (46-73)	54 (25-66)	65 (53-73)	64 (58-72)	61 (46-72)	0·0063
Female sex	124 (40)	31 (26)	26 (36)	20 (38)	5 (18)	206 (35)	0·012
Comorbidities							
Charlson Comorbidity Index	2 (1-5)	2 (1-3)	1 (0-2)	2 (1-4)	2 (1-4)	2 (1-4)	0·0002
History of malignancy	56 (18)	21 (18)	16 (22)	18 (35)	7 (25)	118 (20)	0·074
Immunocompromised	52 (17)	7 (6)	17 (23)	9 (17)	1 (4)	86 (15)	0·0031
Origin of patient							<0·0001
Home	150 (49)	55 (46)	47 (64)	45 (87)	21 (75)	318 (55)	
Long-term care facility	89 (29)	6 (5)	1 (1)	0	3 (11)	99 (17)	
Hospital transfer	68 (22)	59 (49)	24 (33)	6 (12)	3 (11)	160 (28)	
Other[2]	1 (0)	0	1 (1)	1 (2)	1 (4)	4 (1)	
Prior ICU admission	195 (63)	65 (54)	49 (67)	40 (77)	15 (54)	364 (63)	0·042
Patient location at time of first positive culture							0·001
Emergency department	52 (17)	3 (3)	8 (11)	0	1 (4)	64 (11)	
Hospital ward	88 (29)	62 (52)	22 (30)	29 (56)	19 (68)	220 (38)	
Intensive care unit	161 (52)	52 (43)	37 (51)	23 (44)	8 (29)	281 (48)	
Other	7 (2)	3 (3)	6 (8)	0	0	16 (3)	
Days from admission to culture	5 (1-23)	9 (2-26)	21 (2-39)	21 (10-59)	15 (2-23)	8 (1-27)	<0·0001
Hospital-acquired	173 (56)	88 (73)	53 (73)	42 (81)	20 (71)	376 (65)	0·0002
Source of infection							<0·0001
Blood	29 (9)	10 (8)	15 (21)	12 (23)	3 (11)	69 (12)	
Respiratory	194 (63)	94 (78)	30 (41)	31 (60)	9 (32)	358 (62)	
Urine	47 (15)	11 (9)	19 (26)	6 (12)	5 (18)	88 (15)	
Wound	38 (12)	5 (4)	9 (12)	3 (6)	11 (39)	66 (11)	
Pitt Bacteremia Score	4 (2-6)	2 (0-4)	3 (0-6)	2 (1-6)	2 (0-4)	3 (2-6)	<0·0001
Polymicrobial	103 (33)	58 (48)	16 (22)	9 (17)	16 (57)	202 (35)	<0·0001

All data are shown as n (% of total) or median (interquartile range)

[1]*P* values to assess differences between groups. The chi-square test was used for categorical variables and the Kruskal-Wallis test was used for continuous variables.

[2]Other includes patients transferred from a foreign country or from hospice.

Supplemental Table 2. Antimicrobial susceptibilities of carbapenemase-producing vs. non-carbapenemase-producing CRPA isolates based on local laboratory testing.

Antimicrobial Agent	Carbapenemase-producer No. susceptible / No. tested (%)	Non-carbapenemase-producer No. susceptible / No. tested (%)	*P*
Amikacin	77/206 (37%)	500/590 (85%)	<0·0001
Cefepime	14/193 (7%)	289/694 (42%)	<0·0001
Ceftazidime	6/176 (3%)	172/439 (39%)	<0·0001
Ciprofloxacin	12/202 (6%)	256/730 (35%)	<0·0001
Gentamicin	36/177 (20%)	466/693 (67%)	<0·0001
Piperacillin-tazobactam	8/146 (5%)	252/700 (36%)	<0·0001

Only agents for which ≥50% of isolates were tested in each category are included in this analysis.

Supplemental Table 3. CRPA Clonal Group by geographic region and associations with carbapenemases and infection (vs. colonization).

Clonal group (CG)	Total (n=972)	USA (n=527)	China (n=171)	South-Central America (n=127)	Middle East (n=91)	Australia-Singapore (n=56)	Carbapenemases (n)	No. associated with infection
CG235	117 (12)	50 (9)	6 (4)	34 (27)	14 (15)	13 (23)	KPC-2 (25); GES-5 (11); IMP-1 (11); IMP-13 (1); OXA-23 (1); VIM-2 (1); Non-CP (67)	56 (48)
CG111	79 (8)	39 (7)	2 (1)	26 (20)	12 (13)	0	VIM-2 (24); KPC-2+VIM-2 (14); IMP-18+VIM-2 (3); Non-CP (38)	51 (65)
CG298	38 (4)	35 (7)	0	3 (2)	0	0	KPC-2 (2); KPC2+VIM-2 (1); Non-CP (35)	24 (63)
CG463	34 (3)	0	34 (20)	0	0	0	KPC-2 (31); Non-CP (3)	19 (56)
CG253	33 (3)	27 (5)	1 (1)	1 (1)	2 (2)	2 (4)	KPC-2 (2); Non-CP (31)	17 (52)
CG244	30 (3)	17 (3)	7 (4)	3 (2)	2 (2)	1 (2)	VIM-2 (3); KPC-2 (1); Non-CP (26)	16 (53)
CG282	26 (3)	26 (5)	0	0	0	0	Non-CP (26)	15 (58)
CG274	25 (3)	10 (2)	6 (4)	4 (3)	3 (3)	2 (4)	KPC-2 (2); VIM-2 (1); Non-CP (22)	12 (48)
CG308	21 (2)	4 (1)	0	3 (2)	2 (2)	12 (21)	NDM-1 (12); VIM-2 (2); non-CP (7)	12 (57)
CG357	21 (2)	2 (0)	4 (2)	5 (4)	9 (10)	1 (2)	IMP-15 (2); KPC-2+VIM-2 (2); KPC-2 (1); NDM-1 (1); Non-CP (15)	13 (62)
CG179	19 (2)	17 (3)	1 (1)	1 (1)	0	0	KPC-3 (1); VIM-1 (1); VIM-2 (1); VIM-24 (1); Non-CP (15)	7 (37)
CG233	11 (1)	1 (0)	1 (1)	3 (2)	5 (5)	1 (2)	VIM-2 (8); VIM-6 (1); Non-CP (2)	8 (73)
CG654	9 (1)	0	0	7 (6)	2 (2)	0	KPC-2 (6); Non-CP (3)	5 (44)
CG823	9 (1)	0	6 (4)	0	0	3 (5)	VIM-2 (9)	4 (44)
Other	500 (51)	299 (57)	103 (60)	37 (29)	40 (44)	21 (38)		322 (64)

All data are shown as No. (% of total column).
Abbreviations: Non-CP: non-carbapenemase-producing.

Supplemental Table 4. Outcomes of patients infected with CRPA by region using a standardized definition of pneumonia instead of a physician-adjudicated definition.

	USA (n=183)	China (n=70)	South-Central America (n=52)	Middle East (n=26)	Australia-Singapore (n=21)	Total (n=352)	*P*[1]
Mortality[2]							
30-day (primary outcome)	35 (19)	5 (7)	15 (29)	9 (35)	2 (10)	66 (19)	0·0044
90-day	58 (32)	9 (13)	17 (33)	11 (42)	4 (19)	99 (28)	0·010
Length of hospital stay from infection onset	9 (5-20)	15 (7-26)	18 (6-37)	20 (6-33)	36 (14-74)	13 (6-29)	0·0005
DOOR outcome at 30 days[3] (Supplemental Figure 4)							
Alive without events	64 (35)	26 (37)	16 (31)	9 (35)	5 (24)	120 (34)	
Alive with 1 event	52 (28)	24 (34)	5 (10)	2 (8)	2 (10)	85 (24)	
Alive with 2 or 3 events	32 (17)	15 (21)	16 (31)	6 (23)	12 (57)	81 (23)	
Death	35 (19)	5 (7)	15 (29)	9 (35)	2 (10)	66 (19)	
Disposition after discharge							
Home	62 (34)	25 (36)	32 (63)	15 (58)	11 (58)	145 (42)	<0·0001
Long-term care facility	68 (37)	2 (3)	2 (4)	0	4 (21)	76 (22)	
Transfer to another hospital or to a foreign country	2 (1)	25 (36)	0	1 (4)	0	28 (8)	
Hospice	12 (7)	9 (13)	0	0	0	21 (6)	
Death	39 (21)	9 (13)	17 (33)	10 (38)	4 (21)	79 (23)	
Remained in the hospital	0	0	1 (2)	0	2 (10)	3 (1)	
Clinical response	106 (58)	29 (41)	20 (38)	11 (42)	7 (33)	173 (49)	0·015

All data are shown as n (% of total no.) or median (interquartile range).

Abbreviations: DOOR, desirability of outcomes ranking.

[1]*P* values to assess differences among groups. Chi-square was used for categorical variables and Kruskal-Wallis test was used for continuous variables.

[2]Patients who were discharged to hospice were not considered to have died.

[3]The three adverse events assessed by DOOR were: lack of clinical response, lack of discharge within 30 days or readmission within 30 days, and incident renal failure or *Clostridioides difficile* infection.

Supplemental Table 5. Characteristics and outcomes of patients with carbapenemase-producing vs. non-carbapenemase-producing CRPA infections outside of the USA.

	**Carbapenemase producer (n=120)**	**Non-carbapenemase producer (n=153)**	***P***
Patient Characteristic			
Age	58 (36-68)	62 (46-73)	0·096
Female sex	34 (28)	48 (31)	0·59
Geographic region			<0·0001
China	30 (25)	90 (59)	
South-Central America	53 (44)	20 (13)	
Middle East	20 (17)	32 (21)	
Australia-Singapore	17 (14)	11 (7)	
Charlson Comorbidity Index	2 (0-3)	2 (1-3)	0·36
History of malignancy	28 (23)	34 (22)	0·83
Immunocompromised	23 (19)	11 (7)	0·0029
Origin of patient			0·79
Home	77 (64)	91 (59)	
Long-term care facility	5 (4)	5 (3)	
Hospital transfer	37 (31)	55 (36)	
Transfer from foreign country	1 (1)	2 (1)	
Infection Characteristics			
Patient location at time of infection onset			0·19
Emergency department	8 (7)	4 (3)	
Hospital ward	56 (47)	76 (50)	
Intensive care unit	50 (42)	70 (46)	
Other	6 (5)	3 (2)	
Hospital-acquired	88 (73)	115 (75)	0·73
Anatomical source			<0·0001
Blood	25 (21)	15 (10)	
Respiratory	44 (37)	120 (78)	
Urine	36 (30)	5 (3)	
Wound	15 (13)	13 (8)	
Pitt Bacteremia Score	3 (1-6)	2 (0-4)	0·094
Polymicrobial	33 (28)	66 (43)	0·0076
Outcomes			
30-day mortality[1] (primary outcome)	26 (22)	19 (12)	0·041
90-day mortality	33 (28)	28 (18)	0·070
Length of hospital stay from infection onset	17 (7-39)	21 (8-38)	0·18
DOOR at 30 days[2]			
Alive without events	37 (31)	54 (35)	
Alive with 1 event	22 (18)	28 (18)	
Alive with 2 or 3 events	35 (29)	52 (34)	
Death	26 (22)	19 (12)	
Disposition after discharge			0·058
Death	31 (26)	27 (18)	
Home	60 (50)	69 (45)	
Hospice	5 (4)	7 (5)	
Long-term care facility	9 (7)	7 (5)	
Transfer to another hospital or to a foreign country	14 (12)	38 (26)	
Remained in hospital	1 (1)	5 (3)	
Clinical response	47 (39)	61 (40)	0·91

All data are shown as n (% of total no.) or median (interquartile range).
Abbreviations: DOOR, desirability of outcomes ranking.
[1]Patients who were discharged to hospice were not considered to have died.
[2]The three adverse events assessed by DOOR were: lack of clinical response, lack of discharge within 30 days or readmission within 30 days, and incident renal failure or *Clostridioides difficile* infection. The unadjusted DOOR probability estimate of a favorable outcome in CP-CRPA vs. non-CP-CRPA infections was 46% (95% confidence interval: 39%-52%).

Supplemental Table 6. Unadjusted and IPW-adjusted 30-day mortality (%) among patients infected with CP-CRPA vs. non-CP-CRPA stratified by geographic regions (excluding USA patients).

Mortality	China (n=120)			South-Central America (n=73)			Middle East (n=52)			Australia-Singapore (n=28)		
	**CP-CRPA (n=30)**	**Non-CP-CRPA (n=90)**	**Difference (95% CI)**	**CP-CRPA (n=53)**	**Non-CP-CRPA (n=20)**	**Difference (95% CI)**	**CP-CRPA (n=20)**	**Non-CP-CRPA (n=32)**	**Difference (95% CI)**	**CP-CRPA (n=17)**	**Non-CP-CRPA (n=11)**	**Difference (95% CI)**
Unadjusted 30-day mortality	7%	6%	1% (-5%, 7%)	26%	30%	-4% (18%, 11%)	40%	22%	18% (1%, 36%)	12%	9%	3% (-14%, 19%)
IPW-adjusted 30-day mortality	11%	5%	6% (-1, 13%)	25%	26%	-1% (15%, 13%)	42%	22%	20% (2%, 38%)	30%	13%	17% (-5%, 38%)

Abbreviations: CP-CRPA: carbapenemase-producing *Pseudomonas aeruginosa*; IPW: inverse probability weighted.
IPW analysis adjusted for anatomical culture source and age-adjusted Charlson Comorbidity Index score.

Supplemental Table 7. Unadjusted and IPW-adjusted 30-day and 90-day mortality (%) among patients infected with CP-CRPA vs. non-CP-CRPA using a standardized definition of pneumonia instead of a physician-adjudicated assessment.

Mortality Outcome	CP-CRPA (n=94)	Non-CP-CRPA (n=75)	Difference (95% CI)
30-day (unadjusted)	21%	15%	7% (-2%, 15%)
30-day (IPW-adjusted)	21%	16%	5% (-3%, 13%)
90-day (unadjusted)	27%	21%	5% (-4%, 14%)
90-day (IPW-adjusted)	27%	21%	5% (-4%, 15%)

Abbreviations: IPW, inverse probability weighted.

IPW analysis adjusted for geographic region, age-adjusted Charlson Comorbidity Index, patient location prior to hospitalization, immunocompromised status, and anatomical source.

Supplemental Table 8. Multivariate logistic regression model of baseline covariates and their associations with 30-day mortality after CRPA infection (excluding USA patients, with a random effect for study site).

Baseline covariate	Adjusted odds ratio (95% CI)
Carbapenemase (vs. no carbapenemase)	2.09 (0.93-4.70)
Age-adjusted Charlson Comorbidity Index score (per unit increase)	1.07 (0.93-1.22)
Immunocompromised	1.51 (0.52-4.37)
Location of patient other than home prior to hospitalization	1.19 (0.54-2.66)
Anatomical source (vs. wound)	
Blood	8.34 (1.54-45.24)
Respiratory	2.64 (0.53-13.12)
Urine	0.49 (0.057-4.10)

Supplemental Table 9. Multivariate Cox proportional hazards model of baseline covariates and their associations with 30-day mortality after CRPA infection (excluding USA patients).

**Baseline covariate**	**Adjusted hazard ratio (95% CI)**
Carbapenemase (vs. no carbapenemase)	1.41 (0.71-2.80)
Geographic region (vs. South/Central America)	
China	0.17 (0.064-0.43)
Middle East	0.87 (0.41-1.85)
Australia-Singapore	0.36 (0.10-1.26)
Age-adjusted Charlson Comorbidity Index score (per unit increase)	1.07 (0.96-1.19)
Immunocompromised	1.22 (0.54-2.74)
Location of patient other than home prior to hospitalization	1.73 (0.87-3.44)
Anatomical source (vs. wound)	
Blood	6.50 (1.43-29.6)
Respiratory	2.55 (0.58-11.26)
Urine	0.46 (0.062-3.44)

Supplemental Figure 1. **Flow diagram of patients included in the study**.

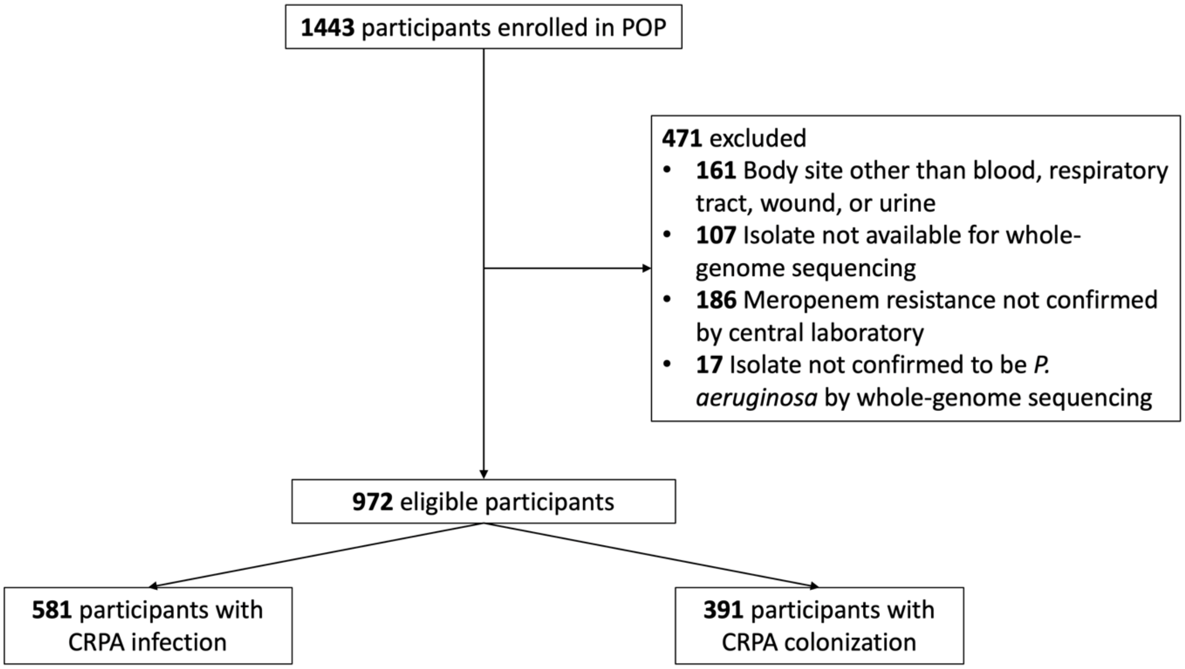

Supplemental Figure 2. Meropenem minimum inhibitory concentration (MIC) distributions of carbapenemase-producing (CP)-CRPA and non-CP-CRPA.

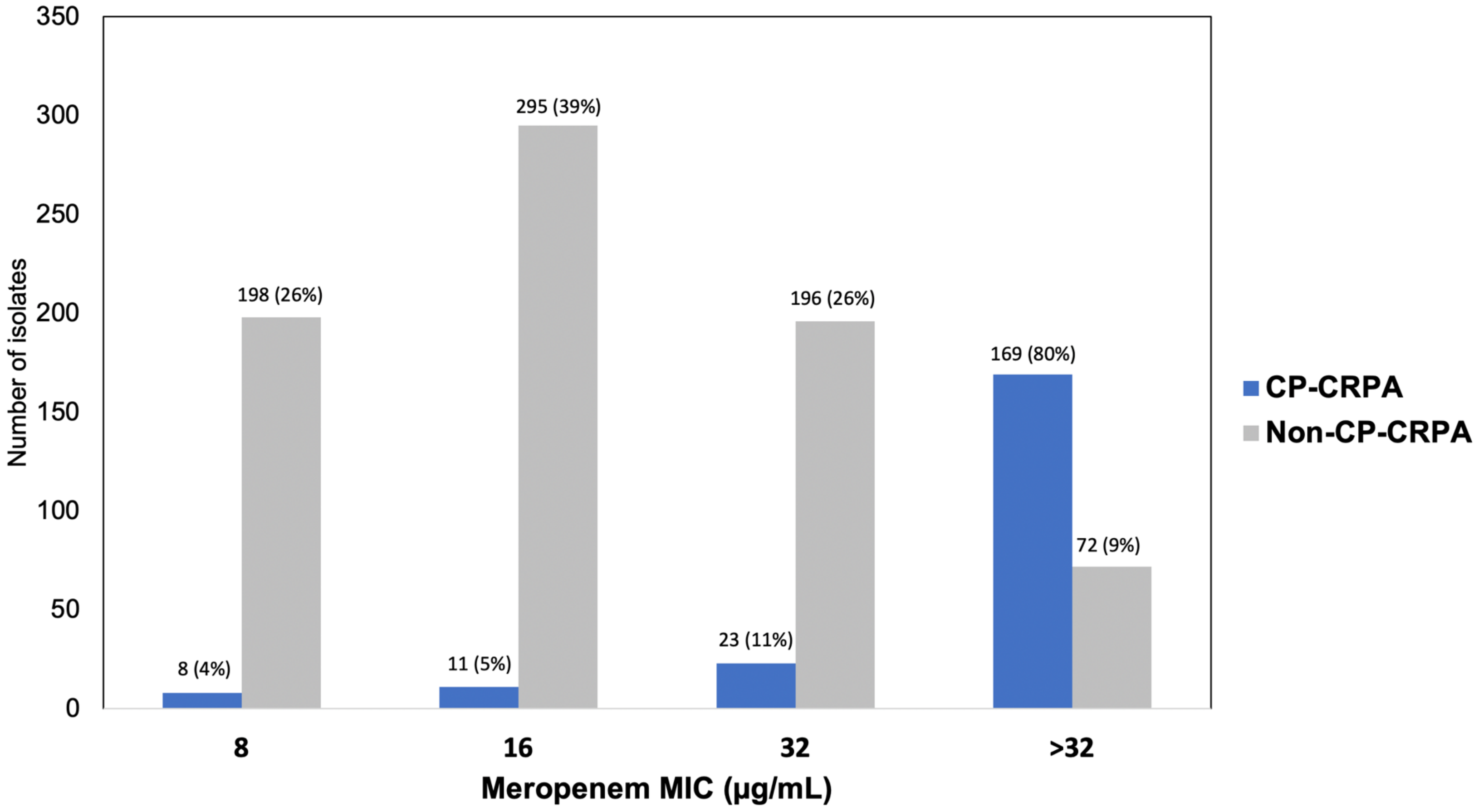

Supplemental Figure 3. DOOR outcomes stratified by culture anatomical source and infection vs. colonization.

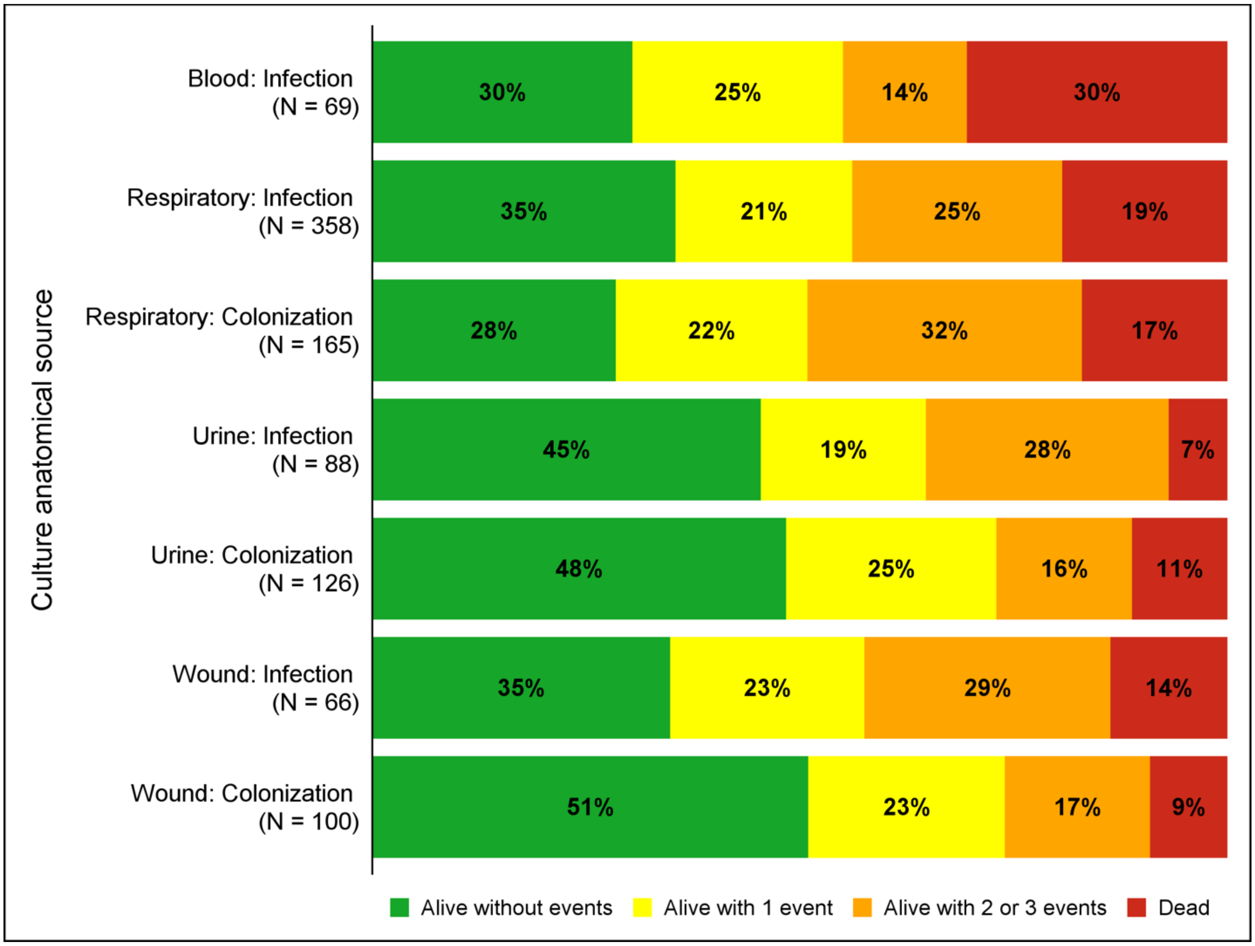

Supplemental Figure 4. DOOR outcomes stratified by geographical region.

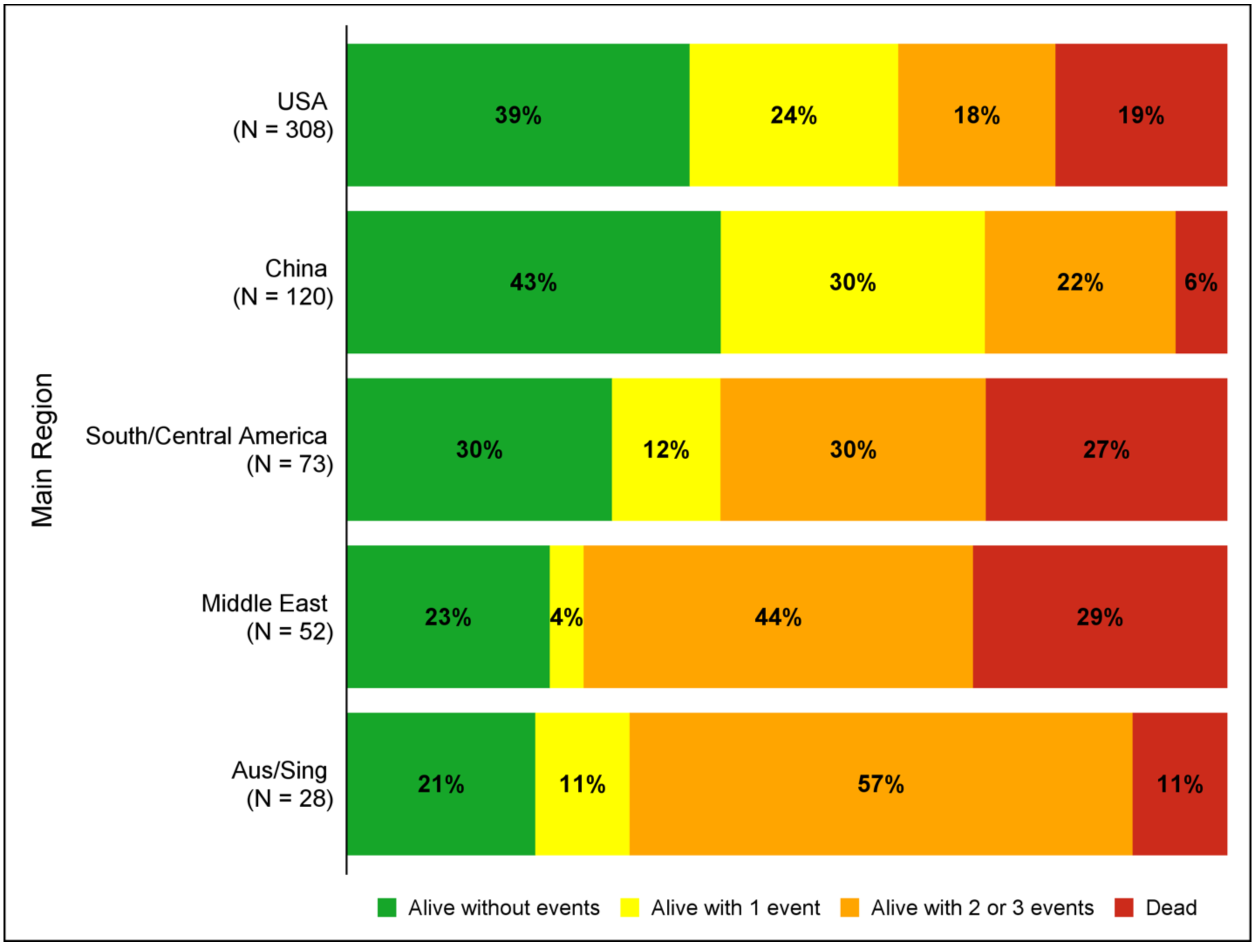

Supplement: 1 [file NIHMS1878924-supplement-1.pdf]
